# CXC ELR-Positive Chemokines as Diagnostic and Prognostic Markers for Breast Cancer Patients

**DOI:** 10.3390/cancers15123118

**Published:** 2023-06-08

**Authors:** Joanna Motyka, Aleksandra Kicman, Monika Kulesza, Sławomir Ławicki

**Affiliations:** 1 Department of Population Medicine and Lifestyle Diseases Prevention, Medical University of Bialystok, 15-269 Bialystok, Poland; joanna.motyka@sd.umb.edu.pl (J.M.);; 2 Department of Aesthetic Medicine, Medical University of Bialystok, 15-267 Bialystok, Poland

**Keywords:** breast cancer, prognosis, diagnosis, ELR-positive, CXC chemokines, expression, concentration, plasma, serum

## Abstract

**Simple Summary:**

Currently, breast cancer diagnostics do not have readily available diagnostic and prognostic tools, especially in the early stages of the disease. Therefore, there is a constant need to search for tumor markers that can improve current diagnostics. One such molecule that may be a potential marker could be among a group of chemokines. In this work, we summarize reports that evaluate the expression and peripheral blood concentration of ELR-positive CXC chemokines as potential markers and prognostic factors for breast cancer.

**Abstract:**

As the most common type of malignant lesison, breast cancer is a leading challenge for clinicians. Currently, diagnosis is based on self-examination and imaging studies that require confirmation by tissue biopsy. However, there are no easily accessible diagnostic tools that can serve as diagnostic and prognostic markers for breast cancer patients. One of the possible candidates for such markers is a group of chemokines that are closely implicated in each stage of tumorigenesis. Many researchers have noted the potential of this molecule group to become tumor markers and have tried to establish their clinical utility. In this work, we summarize the results obtained by scientists on the usefulness of the ELR-positive CXC group of chemokines in ancillary diagnosis of breast cancer.

## 1. Introduction

Breast cancer remains the most prevalent cancer in the world. According to WHO data from the GLOBOCAN database, the global incidence in 2020 reached more than 2.2 million new cases, with mortality estimated at around 685,000 [1]. Breast cancer mortality, estimated by the 5-year survival period of patients varies depending on the disease stage at the time of diagnosis. For stage IV breast cancer, survival rates are below 30%, whereas for stage I cases, they are over 99% [2]. These numbers illustrate the importance of early diagnosis in treating breast cancer.

Current diagnostic methods are primarily based on imaging methods such as ultrasound, X-ray, and magnetic resonance imaging (MRI). Implementing mammography screening in many countries for groups of women with an increased risk of breast cancer, that is, around the age of 50, was a breakthrough in streamlining the diagnostic process. Many studies have shown a significant decrease in the risk of breast cancer mortality after introducing mammography into medical practice [3,4]. However, imaging methods also have limitations. A major one is the sheer size of the lesion found in the breast. In the case of microscopic tumors, it is difficult to determine the potential nature of the lesion. Consequently, imaging results must be correlated with a patient’s clinical examination and relied on for the histopathologic evaluation of the lesion obtained by tissue biopsy [5]. In current medicine, biopsy evaluation is the only method of confirming a cancerous lesion, which is unfortunately invasive.

Circulating cancer biomarkers have received significant attention in recent years. Venous blood is an easily accessible material, and the collection method has been a routine procedure widely used in disease diagnoses. The most frequently determined blood compounds in diagnosing breast cancer are carcinoma antigen 15-3 (CA 15-3), carcinoembryonic antigen (CEA), or carcinoma antigen 27.29 (CA 27.29). However, it should be noted that these molecules have limited sensitivity and specificity, which results in their unsuitability for screening [6]. Due to the limited utility of current markers in the ancillary diagnosis of breast cancer, there is a search for new compounds whose assays could be an adjunctive tool in the diagnosis of this condition. Such compounds could be chemokines [7]. Chemokines in the body form and maintain the immune response, engage in the process of angiogenesis, and determine the chemoattraction and migration of cells that possess chemokine receptors. As a result, these compounds may be involved in each phase of the tumorigenesis process, from the tumor’s initiation and proliferation by local dissemination up to its spread in distant locations. There have also been reports of chemokines’ role in driving resistance to applied treatments [7,8]. For these reasons, chemokines may be helpful in the diagnostic process and act as potential candidates for tumor markers. Apart from circulating molecules’ blood concentrations, many researchers also note that their elevated or reduced tissue expression may have prognostic significance for the course of treatment. They may also correlate with lymph node involvement, the occurrence of distant metastasis, or at least be associated with a specific molecular subtype of breast cancer. This yet-to-be-studied area brings great, but still rather dim, hopes for advancing the diagnostic and prognostic evaluation of breast cancer. In this paper, we introduce and describe the potential use of the ELR-positive CXC group of chemokines in ancillary diagnosis of breast cancer.

## 2. Breast Cancer: A Brief Overview

Breast cancer is a heterogeneous disease. It has high histopathological and molecular diversity, as well as various degrees of cell differentiation. Breast cancer often manifests itself as a palpable lesion in the breast. Other less common symptoms include tightening or pulling of the skin over the lesion, retraction or leakage of the nipple, or, less commonly, a change in breast size, skin color, or enlargement of the axillary lymph nodes (including lymph node engagement with the simultaneous absence of symptoms originating from the breast itself). Such symptoms can simultaneously occur with benign breast lesions; therefore, imaging and histological examinations based on fine- or core-needle biopsy are necessary for identification [9]. 

Based on the histological type, breast lesions are divided into benign tumors, lesions of an indeterminate, borderline, or uncertain nature, carcinoma in situ, or G3 intraepithelial neoplasia, and malignant tumors with distant metastatic foci [10,11] (Supplementary Table S1). Malignant breast cancers are classified into two major groups: carcinomas and sarcomas. Carcinomas are the most common breast tumors and originate from the epithelial cells of the breasts’ lobules or ducts [11]. Breast cancer can be classified molecularly or by staging systems. Particularly relevant is the molecular classification that divides breast cancer into four major subtypes: Luminal A, Luminal B, HER2-enriched (HER2+), and triple-negative breast cancer (TNBC) [12,13]. Segregation into each subtype is based on the expressions of estrogen receptor (ER), progesterone receptor (PR), human epidermal growth factor receptor 2 (HER2), and cell proliferation factor (Ki-67) [14].

**Figure 1:**
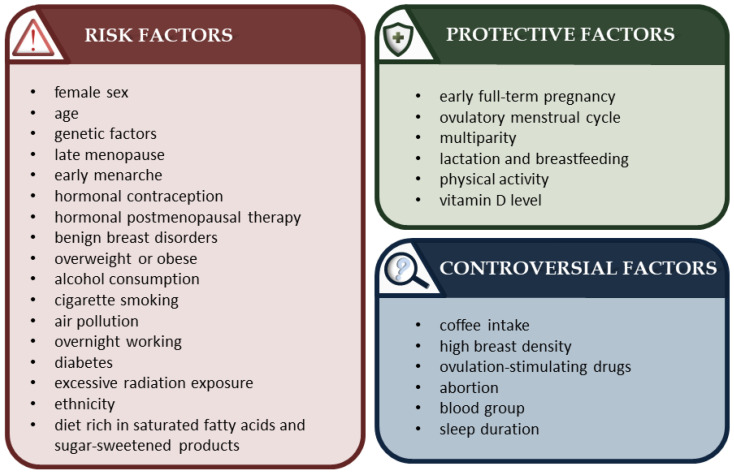
Breast cancer risk and protective factors [9,11,25,26,27].

The pathogenesis of breast cancer is a complex process that remains incompletely studied. Although much is known about risk factors and protective factors, some aspects are still controversial. A diagram containing various factors is shown in Figure 1. 

The biochemical diagnosis of breast cancer has mostly supportive applications and generally uses cancer antigen 15-3 (CA 15-3), cancer-embryonic antigen (CEA), and cancer antigen 27.29 (CA 27.29) [15,16,17,18]. However, the previously mentioned low sensitivity and specificity of these compounds preclude their use in screening [19,20]. In addition, these markers have low concentrations at the early stages of breast cancer, which decreases their screening utility. Importantly, serum concentrations of CA 15-3, CEA, and CA 27.29 are affected by several pathological phenomena that coexist with cancerous lesions in the breast. These include, among others, liver, lung, and endocrine diseases [21,22,23,24]. However, the aforementioned compounds have been found to have clinical utility in monitoring the effectiveness of therapy and detecting early recurrence or the presence of metastases, especially in cases where radiological diagnoses cannot be used [6].

## 3. An Outline of the CXC Chemokine Group

CXC chemokines belong to a family of chemotactic cytokines comprising around 50 proteins. They consist of four cysteine residues forming two disulfide bridges. The first two cysteine residues are separated by a single amino acid. As previously mentioned, chemokines are involved in controlling many processes, both physiological and pathological. One of their most important functions is regulating immune balance and directing the immune response [28,29]. In addition, CXC group chemokines are among the key elements regulating inflammatory and angiogenic processes. Depending on whether a 3-amino-acid glutamine–leucine–arginine motif (ELR motif) is present at the N-terminus of the chain, the CXC group of chemokines is divided into two subgroups: ELR+ promoting angiogenesis and ELR- with angiostatic properties. It is important to note that CXCL12, which belongs to the ELR- subgroup, is an exception to ELR motif dependence for angiostatic properties, and is instead involved in promoting angiogenesis through its interaction with receptors.

Three mechanisms may involve chemokines in tumorigenesis:

1. Control of angiogenesis—Allows tumor growth and metastasis by providing easy access to oxygen and nutrients.
2. Immune regulation—Controls the influx of leukocytes into the tumor microenvironment.
3. Modification of the functioning of cancer cells—Interacts with chemokine receptors and triggers intracellular signaling pathways.

These mechanisms can act in a procarcinogenic manner, e.g., by stimulating angiogenesis, the influx of lesion-promoting leukocytes, or altering the biological profile of the cell (increased proliferation, avoidance of apoptosis, and decreased adhesion to the microenvironment). However, they can also act in an anticarcinogenic manner by inhibiting angiogenesis and stimulating non-specific anti-tumor immunity through an influx of leukocytes [8,28,29,30,31]. Dysregulation of chemokine expression in the tumor microenvironment can also direct tumor cells to other locations and promote their spread in the body.

The tumor microenvironment and its components, including growth factors [32,33,34], chemokines [33,35,36,37], or matrix metalloproteinases [38,39,40], are of scientific interest for their potential diagnostic and prognostic factors and as potential therapeutic targets. The breast tumor stroma, however, is abundant in fibroblasts, which are one of the main sources of chemokines. Other sources of these molecules include leukocytes, epithelial, endothelial, and mesenchymal cells, including tumor-altered cells [7,28,41,42]. Therefore, focusing on this group of molecules in the breast cancer diagnostic process seems natural.

## 4. CXC ELR-Positive Chemokines as Biomarkers of Breast Cancer

The issues discussed in the current chapter are summarized in Table 1 at the end of the article.

### 4.1. CXCL1

Examination of CXCL1 tissue expression by immunohistochemical (IHC) staining showed an increased expression of this chemokine relative to normal breast tissue [43,44]. On the contrary, data from about 3000 patients from The Cancer Genome Atlas (TCGA) database showed overexpression of CXCL1 mRNA in only 4–7% of breast cancer (BC) patients. The other cases had significantly lower expression than healthy tissue, with this overexpression appearing more frequently in TNBC [45,46,47,48,49,50]. Furthermore, Bild et al. and Narita et al. noted that higher CXCL1 expression was found in TNBC tissues (*p* = 0.0005 [51], *p* < 0.05 [52]). Pawitan et al. showed that an increase in CXCL1 expression was significantly associated with the presence of mutations in the BRCA1 gene (*p* = 0.0035), which is commonly associated with TNBC subtypes [53]. Additionally, higher CXCL1 mRNA expression was noted in ER-negative breast cancer types relative to ER-positive samples (*p* < 0.0001) in data from the Oncomine and TCGA databases [54,55,56]. Yang et al. also showed significantly higher CXCL1 expression in ER-negative breast cancer lines BT-549 and MDA-MB-468 than in ER-positive lines (MCF-7, T47D, ZR-75-1) [56]. High-tissue CXCL1 expression correlated positively with tumor size (*p* = 0.04 [36]), TNM staging (*p* < 0.001 [45]; *p* = 0.002 [57]), positive lymph nodes (*p* = 0.001) [45], degree of lymph node infiltration (*p* = 0.016) [45], the occurrence of distant metastases (*p* = 0.015 [36], *p* < 0.05 [57]), shorter overall survival (*p* < 0.001 [45]; *p* = 0.0186 [55]), and shorter relapse-free survival (*p* = 0.0442 [55]; *p* = 0.017 [57]; *p* = 0.01 [47]). However, Zou et al.’s study showed different results. They studied the correlation between CXCL1 expression and prognostic factors and found no correlation between ER, PR, HER2, or Ki 67 receptor status, tumor size, and lymph node involvement. However, they noted a correlation between high CXCL1 expression and higher tumor grade, metastases, and shorter relapse-free survival [43]. Hozhabri et al. also confirmed increased CXCL1 mRNA expression with tumor grade growth, with grade 3 reaching the highest level and grade 1 (*p* < 0.0001 [50]) for the lowest level. Interestingly, in Li et al.’s study, CXCL1 expression was not associated with overall survival for the entire study group [47,48,49,50]. By contrast, its higher expression correlated with longer relapse-free survival (*p* < 0.001) in the luminal-only breast cancer group [48].

Part of the findings were also related to data obtained from blood tests. Ma et al. [36] noted higher CXCL1 serum levels among breast cancer patients than healthy women (*p* = 0.011). In our own study, we did not compare the CXCL1 plasma concentrations between luminal breast cancer patients and healthy women [35]. Moreover, in women with benign breast lesions, plasma CXCL1 concentrations also remained at similar levels to cancer patients and healthy women [35]. High plasma levels of CXCL1 along with TGF-β in patients with metastatic breast cancer were also associated with increased detection of circulating tumor cells (TGF-β *p* < 0.0001; CXCL1 *p* = 0.02) and shorter overall survival (CXCL1 *p* = 0.05; CXCL1+ TGF-β *p* = 0.001) [33].

### 4.2. CXCL2 and CXCL3

CXCL2, similar to CXCL1, showed increased mRNA expression in only about 7% of primary tumors, whereas, in secondary tumors, the expression reached almost 20% [44,57]. However, the TCGA and Oncomine dataset showed that CXCL2 expression, alongside CXCR3 expression, is significantly lower in healthy tissues than in breast cancer (*p* < 0.001 [47,48,49,58]; *p* < 0.0001 [50]). CXCL2 and CXCL3 expression follow a similar pattern to CXCL1, and their overexpression in TNBC compared to ER-positive lines can be observed [47,48,52,57,58]. An et al. also examined the expression’s effect on prognostic factors based on available databases. According to two databases, GSE3494-GPL96 and GSE1456-GPL96, high CXCL2 expression was associated with longer relapse-free survival (*p* = 0.002; *p* = 0.003, respectively). According to the GSE3143 dataset, CXCL2 expression was inversely correlated with length of time to overall survival (*p* = 0.017) [58]. The survival analysis results based on the Kaplan–Meier plotter database, however, opposed those obtained by the GSE3143 dataset, where, ultimately, higher CXCL2 expression correlated with longer overall survival (*p* < 0.001 [58]; *p* = 0.021 [48,49]) and longer relapse-free survival (*p* = 0.00034 [50]; *p* < 0.001 [47]). Additionally, higher CXCL2 expression correlated inversely with metastasis (*p* = 0.002), resulting in a better prognosis [47]. 

Despite the similarities between CXCL2 and CXCL3 expression patterns, higher CXCL3 expression appears to be an unfavorable prognostic factor. Increased CXCL3 expression was found in aggressive and metastatic cancer cells [57,59] and was associated with higher tumor grades (*p* < 0.001) [50], shorter overall survival (*p* = 0.042) [48,49], and shorter relapse-free survival (*p* = 0.038) [57]. However, studies by Chen et al. and Hozhabri et al. showed an inverse relationship for CXCL3, where its higher expression was associated with longer relapse-free survival (*p* = 0.015 [47]; *p* = 0.016 [50]) and did not affect overall survival [47,50].

### 4.3. CXCL5

Studies using data from the Oncomine database showed significantly lower CXCL5 mRNA expression in breast cancer tissue than in healthy breast tissue (*p* < 0.05) [47,48,49]. However, based on data from the TCGA and Genotype-Tissue Expression databases, expression levels were not significantly different [48,49]. Overexpression of CXCL5 in TNBC compared to ER+ subtypes was also noted in molecular subtype analyses of breast cancer [48,49,50,55,57]. Higher expression was also shown in metastatic cells and grade 3 tumor cells than in grade 1 primal tumor cells (*p* < 0.05 [57]; *p* < 0.0001 [50]). CXCL5 expression showed no correlation with overall survival [47,48,49,50]. Depending on the sources, higher CXCL5 expression did not correlate [48,49,57] or positively correlate (*p* = 0.008 [47]; *p* = 0.04 [50]) with longer relapse-free survival. Li et al. [60] had contradictory findings based on the Gene Expression Omnibus database (GSE12276, GSE2603, GSE2034, and GSE5327) and determined that low CXCL5 expression was associated with better outcomes for metastasis-free survival (*p* = 0.021). Additionally, by examining the protein’s tissue expression by IHC staining, CXCL5 protein expression was significantly higher in breast cancer tissues than in tissues of healthy controls (*p* < 0.05) [60]. 

Li et al. [60] obtained similar results when serum concentrations were determined. Higher concentrations were obtained in breast cancer patients than in the serum of healthy controls (*p* < 0.001) [60]. Li et al. also determined the potential of serum CXCL5 concentrations as a diagnostic marker by obtaining a diagnostic sensitivity of 65.3%, a diagnostic specificity of 60%, and a diagnostic test power of AUC = 0.6970 [60]. Wang et al. also studied the serum concentration of CXCL5 and found that it did not differ between breast cancer patients and patients with benign and proliferative breast lesions. However, the concentration differed by tumor size (*p* < 0.001) and positively correlated with Ki-67 expression levels (*p* = 0.027) [37].

### 4.4. CXCL6 and CXCL7

Bièche et al.’s study of CXCL6 mRNA expression in breast cancer, as well as data analyses from the TCGA, Oncomine, and Genotype-Tissue Expression databases, showed no differences between tumor and physiological breast tissues [48,49,57]. However, expression was higher in the TNBC molecular subtype than in ER+ subtypes (*p* < 0.05) [48]. However, Chen et al. and Hozhabri et al. showed significantly lower CXCL6 expression in breast cancer than in normal tissues (*p* < 0.05) [47,50]. They noted that the CXCL6 expression level was significantly higher in metastatic cancer cells and grade 3 tumor cells than in low graded cells but showed no correlation with overall survival (*p* < 0.05 [57]; *p* < 0.001 [50]). Chen et al. and Hozhabri et al. also noticed this lack of correlation but managed to show a positive correlation between CXCL6 expression and relapse-free survival (*p* < 0.001 [47]; *p* = 0.00012 [50]). On the other hand, other studies revealed that CXCL6 overexpression was associated with longer overall survival (*p* = 0.036) [48,49].

CXCL7 mRNA expression levels, depending on the database, showed lower levels in cancerous tissue than in physiological breast tissue (*p* < 0.05) (Oncomine and UALCAN databases) or no difference between levels in these tissues (TCGA and GTEx databases) [47,48,49,50]. However, CXCL7 expression was unaffected by receptor status [47,48]. CXCL7 expression correlated with longer relapse-free survival (*p* = 0.014 [47]; *p* = 0.018 [50]) but not with overall survival [47,48,49,50]. Wang et al. obtained inverse correlations, where stage III had higher expression than stage I (*p* < 0.05) or stage II (*p* < 0.001) breast cancer tissue and correlated with worse overall survival (*p* = 0.0017) [61]. 

Wang et al. [37] found that CXCL7 serum levels were negatively correlated with Ki 67 expression levels (*p* = 0.042). However, serum levels did not differ between malignant, benign, and proliferative breast lesions [37]. Kosir and Ju [62] also examined CXCL7 serum levels and noted that they were significantly higher in breast cancer patients than in healthy individuals (*p* < 0.05). Based on 23 pairs of serous specimens, they also assessed that, relative to preoperative levels, postoperative CXCL7 levels decreased significantly (*p* < 0.05) and reached levels comparable to healthy controls [62].

### 4.5. CXCL8

Depending on the source, CXCL8 mRNA in breast cancer is overexpressed (*p* < 0.05) [47,52], underexpressed (*p* < 0.001 [50]), or not differentially expressed [48,49] in healthy tissue. Higher expression has been found in ER-negative types (*p* = 0.019 [52]; *p* < 0.002 [55]; *p* < 0.05 [63]), TNBC (*p* < 0.0001 [47]; *p* < 0.01 [48,49]) and HER2+ (*p* = 0.0009 [47]; *p* < 0.05 [48,49]; *p* < 0.0001 [50]) than in ER+ types of breast cancer. CXCL8 expression has varied by breast cancer stage (*p* = 0.00561) [48,49] and is higher in metastatic tissues and grade 3 tumor cells than in grade 1 and 2 tumor cells (*p* < 0.05 [57]; *p* < 0.0001 [50]).

Higher CXCL8 expression correlated with shorter overall survival (*p* = 0.022 [63]; *p* = 0.0003 [47]; *p* < 0.0001 [48,49,50]), shorter relapse-free survival (*p* < 0.0001 [47,48,49,50]; *p* = 0.009 [57]), and metastatic recurrence (*p* < 0.0001 [57]). The worst prognosis was shown for ER-type breast cancer with high CXCL8 expression and significantly shorter overall survival (*p* < 0.001 [63]) and relapse-free survival (*p* = 0.036 [48,49]). Low CXCL8 mRNA expression indicated a higher chance of 10-year survival with sensitivity, specificity, and test power equal to 63.16%, 65.12%, and AUC = 0.6328, respectively. The tissue expression of CXCL8 protein was higher in breast cancer than in healthy tissues (*p* < 0.05 [48,49]). It was also higher in ER- status tissues than in ER+ tissues (*p* = 0.006 [64]). Higher CXCL8 protein expression directly correlated with shorter relapse-free survival (*p* < 0.001 [64]) and negatively correlated with ER expression (*p* = 0.02 [64]). Kamalakar et al. did not determine a correlation between ER status and CXCL8 tissue expression [65], but this may be due to their small sample size. However, no correlation was shown between CXCL8 tissue expression and age, menopausal status, tumor size, or tumor grade [64].

Several scientific papers have also reported increased serum CXCL8 (*p* = 0.047 [66]; *p* < 0.001 [67]; *p* < 0.001 [68]) or plasma levels (*p* < 0.001 [52]; *p* = 0.005 [35]) in patients with early (*p* = 0.002) and advanced stages (*p* = 0.001) of breast cancer [69] compared with healthy women. Additionally, higher concentrations relative to healthy controls were shown for the ER+ (*p* = 0.021), PR+ (*p* = 0.039), and TNBC (*p* = 0.046) subgroups of breast cancer [68]. Women with benign breast lesions also showed higher levels of CXCL8 than healthy women (*p* < 0.001 [52]; *p* = 0.033 [35]; *p* < 0.001 [68]), but only one paper showed significant differences between malignant and benign lesions (*p* < 0.001 [68]). Wang et al. also noted significant differences between benign lesions and in situ versus invasive cancer (*p* = 0.006), but no post hoc evaluation was performed to define the differences between the groups [37]. However, they did evaluate the differences between the in situ group and the invasive type, obtaining higher concentrations for the in situ type (*p* = 0.002). Based on the results of binary logistic regression analysis, they found that serum CXCL8 concentration was a predictor of differentiation between the benign lesion group (*p* = 0.024), breast cancer group (*p* = 0.011), and healthy controls [37]. According to these results, CXCL8 may be a useful diagnostic marker for breast cancer, which we also investigated in a different paper. CXCL8 concentration showed higher sensitivity (70%), positive predictive value (77.78%), negative predictive value (50%), diagnostic test power (AUC = 0.6410), and similar specificity (60%) than CA 15-3 (55%; 75.34%; 41.56%; AUC = 0.6300; 64%, respectively) [35]. A panel of CXCL8 and CA 15-3 combined increased the sensitivity of the test to 88%, the negative predictive value to 61.29%, and the diagnostic power of the test to AUC = 0.6582 with a high positive predictive value (73.95%) and a decrease in specificity (38%) [35]. Khalaf et al. also evaluated the diagnostic potential of CXCL8 levels. CXCL8 concentration differentiated a group of breast cancer patients from healthy controls with 95.6% sensitivity, 95% specificity, and a diagnostic test power equal to AUC = 0.998. In a test to differentiate benign lesions from healthy individuals, CXCL8 achieved sensitivity, specificity, and diagnostic test power equal to 82.1%, 75%, and AUC = 0.804 [68], respectively. A combination of three chemokines, CXCL8, CXCL9, and CCL22, was evaluated by Narita et al. as a discriminating tool for healthy individuals and those with breast cancer, obtaining a high value of AUC = 0.7771 [52]. These papers vary notably in their suggested diagnostic utility. However, the size of the groups and their specification should be considered. In our work [35], we focused exclusively on luminal subtypes of breast cancer, where the final count of breast cancer patients was 100 and 50 in the group of women with benign lesions and controls, respectively. By contrast, Khalaf et al. studied [68] 45 women with breast cancer, 25 women with benign lesions, and 20 healthy controls. The 45 women with breast cancer included all possible molecular subtypes of breast cancer, which, given the differences in concentration levels based on receptor expression, may have been a key impact on the results.

Reports on CXCL8 concentrations’ behavior in relation to receptor status and clinical features are often contradictory. A few works showed that higher CXCL8 concentrations were present in patients with ER- breast cancer than ER+ types (*p* < 0.0001 [67]; *p* = 0.012 [52]), HER2+ breast cancer type compared to HER2- (*p* < 0.001 [67]), and the TNBC subtype compared to non-TNBC (*p* = 0.001 [68]), but showed no difference in concentration for the PR receptor [67]. Studies by Tiainen et al., Wang, and Benoy showed no correlation between hormone receptor status and CXCL8 concentrations [35,66,68,70]. However, Koning et al. showed that CXCL8 levels were higher for PR- than PR+ breast cancer (*p* = 0.033 [71]; *p* = 0.019 [52]) among patients with no circulating tumor cells (*p* = 0.017) or grade 3 breast cancer (*p* = 0.03) [71]. Higher CXCL8 levels were associated with higher stages (*p* < 0.0001 [67]; *p* < 0.001 [66]), lymph node status (*p* < 0.0068 [66]), and presence of metastasis (*p* < 0.05 [65,67]; *p* = 0.002 [66]). CXCL8 levels did not correlate with age [66], menopause status [66,70], number of metastatic lesions [66,70], presence of visceral disease [66,70], grade [37], or occurrence of recurrence [37,66]. Among patients with current metastases, higher CXCL8 levels were associated with increased tumor load (*p* < 0.0001) and more rapid growth (*p* = 0.0114) [66]. Additionally, the correlation between CXCL8 and NTx levels indicated a marker of bone resorption (*p* < 0.05), confirming this chemokine’s involvement in bone metastasis [65]. Serum CXCL8 concentrations were also an independent prognostic marker, indicating shorter overall survival for patients with higher CXCL8 concentrations (*p* = 0.0045 [66]; *p* = 0.023 [70]). Low CXCL8 concentrations in patients after a course of chemotherapy were also associated with longer overall survival (*p* < 0.001) [70]. CXCL8 increased (*p* < 0.05) for patients whose duration of chemotherapy was prolonged, suggesting its involvement in cytostatic drug resistance [72].

## 5. Conclusions

Breast cancer is the most common malignancy affecting women. This disease is molecularly diverse but has a good prognosis if detected early enough. It is estimated that the 5-year survival rate of patients with stage I breast cancer is as high as 99%. A breast cancer diagnosis is primarily based on imaging examinations such as ultrasound, X-ray, or MRI, which have numerous limitations. Furthermore, the obtained results are confirmed invasively via biopsy. These methods translate into a longer diagnosis time, thus worsening the prognosis for women. There is considerable hope in new ancillary breast cancer diagnosis methods, which include the non-invasive determination of tumor markers from peripheral blood. Potential markers for breast cancer diagnosis include chemokines. This article summarizes existing knowledge about the roles of CXCL ELR-positive chemokines CXCL1, CXCL2, CXCL3, CXCL5, CXCL6, CXCL7, and CXCL8 as peripheral blood and tissue markers in the diagnosis and prognosis of women with breast cancer.

Chemokines have been shown to mediate the pathogenesis of breast cancer. However, data on their potential use in diagnosing the disease are conflicting or contradictory. Although all CXCL ELR-positive chemokines are expressed in cancerous breast tissue, their expression may be elevated or downregulated compared to normal tissue. In addition, the mRNA levels of these chemokines are not always associated with the clinical features of breast cancer, or the data obtained by different research teams are mutually exclusive. We reached similar conclusions after analyzing data on CXCL ELR-positive chemokines’ potential as markers in peripheral blood. Nevertheless, several compounds in this group show considerable potential as tissue or peripheral blood markers.

Establishing the unequivocal importance of CXCL ELR-positive chemokines in breast cancer requires more research. However, we believe that several compounds in this group show high potential. Based on our analysis of accumulated data, CXCL8 has preliminary high potential as a new diagnostic and prognostic marker, while CXCL2 and CXCL6 can be considered as prognostic markers. In the case of CXCL1 and CXCL5, however, we cannot determine their usefulness due to insufficient information.

Regarding TNBC, characterized by a particularly unfavorable prognosis, CXCL1, CXCL2, CXCL3, and CXCL8 are found to be strongly overexpressed relative to luminal types of breast cancer, suggesting that these chemokines can be used to differentiate cancer subtypes or as potential indirect therapeutic targets. The first studies of targeted therapies against individual chemokines did not show the expected efficacy [73,74] due to the multiligand character of their receptors. However, targeting the receiving point in the ligand-receptor signaling axis or using modified immune cells in therapy may result in the expected therapeutic effect, which may, unfortunately, result in severe side effects on the organism’s healthy cells and tissue [75,76]. Nevertheless, new strategies such as modified cell therapy are worth the interest and may signify a breakthrough in treating breast cancer, especially the TNBC subtype.

**Table 1:** Summary table of ELR-positive CXC chemokines prognostic and diagnostic potential with dissected molecule source (circulating protein/tissue protein/tissue mRNA level).

CXCL1									
Source	mRNA			Tissue protein			Blood circulating protein		
Expression/Concentration towards HC	Up	No diff.	Down	Up	No diff.	Down	Up	No diff.	Down
Refs.	[45,48] *	[51]	[45,46,47,48,49,50]	[43,44]	[49]		[36]	[35] **	
Expression/Concentration towards BLC	Up	No diff.	Down	Up	No diff.	Down	Up	No diff.	Down
Ref.								[35] **	
Differences of level between BC subtypes	Yes		No	Yes		No	Yes		No
Refs.	[45,48,50,51,52,54,55,56]		[47]			[43]			
RFS when upregulated	Longer	UA	Shorter	Longer	UA	Shorter	Longer	UA	Shorter
Ref.	[47], [48] **	[48] ***, [50]	[43,45,55,57]						
OS when upregulated	Longer	UA	Shorter	Longer	UA	Shorter	Longer	UA	Shorter
Refs.		[47,48,49,50]	[45,55]						[33]
**CXCL2**									
Source	mRNA			Tissue protein			Blood circulating protein		
Expression/Expression/Concentration towards HC	Up	No diff.	Down	Up	No diff.	Down	Up	No diff.	Down
Refs.			[39,47,48,50,58]		[49],				
Expression/Concentration towards BLC	Up	No diff.	Down	Up	No diff.	Down	Up	No diff.	Down
Ref.									
Differences of level between BC subtypes	Yes		No	Yes		No	Yes		No
Refs.	[47,48,50,52,57,58]								
RFS when upregulated	Longer	UA	Shorter	Longer	UA	Shorter	Longer	UA	Shorter
Ref.	[47], [48] **, [50,58]	[48] ***	[58]						
OS when upregulated	Longer	UA	Shorter	Longer	UA	Shorter	Longer	UA	Shorter
Refs.	[48,49,58]	[47,50]							
**CXCL3**									
Source	mRNA			Tissue protein			Blood circulating protein		
Expression/Concentration towards HC	Up	No diff.	Down	Up	No diff.	Down	Up	No diff.	Down
Refs.			[39,47,48,49,50,58]		[49],				
Expression/Concentration towards BLC	Up	No diff.	Down	Up	No diff.	Down	Up	No diff.	Down
Refs.									
Differences of level between BC subtypes	Yes		No	Yes		No	Yes		No
Refs.	[48,50,52,57,58]		[47]						
RFS when upregulated	Longer	UA	Shorter	Longer	UA	Shorter	Longer	UA	Shorter
Refs.	[47], [58] ** [50]	[48] ***	[57]						
OS when upregulated	Longer	UA	Shorter	Longer	UA	Shorter	Longer	UA	Shorter
Refs.		[47,50]	[48,49]						
**CXCL5**									
Source	mRNA			Tissue protein			Blood circulating protein		
Expression/Concentration towards HC	Up	No diff.	Down	Up	No diff.	Down	Up	No diff.	Down
Refs.		[50],	[47,48,49]	[60]	[49]		[60]		
Expression/Concentration towards BLC	Up	No diff.	Down	Up	No diff.	Down	Up	No diff.	Down
Ref.								[37]	
Differences of level between BC subtypes	Yes		No	Yes		No	Yes		No
Refs.	[48,50,55,57]		[47]						
RFS when upregulated	Longer	UA	Shorter	Longer	UA	Shorter	Longer	UA	Shorter
Refs.	[47,50]	[48,57]							
OS when upregulated	Longer	UA	Shorter	Longer	UA	Shorter	Longer	UA	Shorter
Refs.		[47,48,49,50]							
**CXCL6**									
Source	mRNA			Tissue protein			Blood circulating protein		
Expression/Concentration towards HC	Up	No diff.	Down	Up	No diff.	Down	Up	No diff.	Down
Refs.		[48,49,57]	[47,50,58]		[49]				
Expression/Concentration towards BLC	Up	No diff.	Down	Up	No diff.	Down	Up	No diff.	Down
Ref.									
Differences of level between BC subtypes	Yes		No	Yes		No	Yes		No
Refs.	[48,50]		[47]						
RFS when upregulated	Longer	UA	Shorter	Longer	UA	Shorter	Longer	UA	Shorter
Refs.	[47,50]	[48]							
OS when upregulated	Longer	UA	Shorter	Longer	UA	Shorter	Longer	UA	Shorter
Refs.	[48,49]	[47,50]							
**CXCL7**									
Source	mRNA			Tissue protein			Blood circulating protein		
Expression/Concentration towards HC	Up	No diff.	Down	Up	No diff.	Down	Up	No diff.	Down
Refs.		[48,49]	[47,48,49,50]		[49]		[62]		
Expression/Concentration towards BLC	Up	No diff.	Down	Up	No diff.	Down	Up	No diff.	Down
Ref.								[37]	
Differences of level between BC subtypes	Yes		No	Yes		No	Yes		No
Refs.			[47,48]						
RFS when upregulated	Longer	UA	Shorter	Longer	UA	Shorter	Longer	UA	Shorter
Refs.	[47,50]	[48]							
OS when upregulated	Longer	UA	Shorter	Longer	UA	Shorter	Longer	UA	Shorter
Refs.		[47,48,49,50]	[61]						
**CXCL8**									
Source	mRNA			Tissue protein			Blood circulating protein		
Expression/Concentration towards HC	Up	No diff.	Down	Up	No diff.	Down	Up	No diff.	Down
Refs.	[47,51]	[47,48,49,50,51]	[50]	[49]			[35] **, [52,66,67,68,69]		
Expression/Concentration towards BLC	Up	No diff.	Down	Up	No diff.	Down	Up	No diff.	Down
Refs.							[37,68]	[35] **, [52]	
Differences of level between BC subtypes	Yes		No	Yes		No	Yes		No
Refs.	[47,48,50,51,52,55,63]			[64]		[65]	[52,67],		[35] **, [66,68,70]
RFS when upregulated	Longer	UA	Shorter	Longer	UA	Shorter	Longer	UA	Shorter
Refs.		[48] **	[47], [48] ***, [50]			[64]		[37,66]	
OS when upregulated	Longer	UA	Shorter	Longer	UA	Shorter	Longer	UA	Shorter
Ref.			[47,48,49,50,63]						[66,70]

BLC—benign lesion condition, HC—healthy condition, ND—no data, No diff.—no differences, OS—overall survival, Ref.—references, RFS—relapse free survival, red color *—for TNBC only, blue color **—for luminal BC only, green color ***—for ER-negative BC only, UA—unaffected.

## Data Availability

Publicly available datasets were analyzed in this study. The data that support the findings of this study are available in PubMed and Google Scholar search.

## Supplementary Materials

The following supporting information can be downloaded at: https://www.mdpi.com/article/10.3390/cancers15123118/s1, Table S1: Histological classification of breast tumors. References [9,10] are cited in the Supplementary Materials.
