# Supplementary material for: CXC ELR-Positive Chemokines as Diagnostic and Prognostic Markers for Breast Cancer Patients
Source: Cancers (Basel). 2023 Jun 8;15(12):3118. doi: 10.3390/cancers15123118 (PMC10296565; doi:10.3390/cancers15123118)
Supplement: Supplementary file 1 [file cancers-15-03118-s001.zip › cancers-2319875-supplementary.pdf]

Supplementary Table S1. Histological classification of breast tumors [9,10].

Hyperplasia of breast tissue	Benign tumor	Unspecified, borderline or uncertain behavior	Carcinoma in situ or G3 intraepithelial neoplasia	Malignant tumors
		**Epithelial tumors**		
Non-invasive lobular neoplasia:<br>– atypical lobular hyperplasia<br>Benign epithelial proliferations and precursor:<br>– usual ductal hyperplasia<br>– columnar cell lesions including flat epithelial atypia<br>– atypical ductal hyperplasia<br>– adenosis and benign sclerosing lesions:<br>– sclerosing adenosis<br>– microglandular adenosis<br>Radial scar / complex sclerosing lesion	Epithelial - myoepithelial tumors:<br>– pleomorphic adenoma<br>– adenomyoepithelioma NOS<br>Papillary neoplasms:<br>– intraductal papilloma<br>Adenomas:<br>– tubular adenoma NOS<br>– lactating adenoma<br>– duct adenoma NOS<br>Adenosis and benign sclerosing lesions:<br>– apocrine adenoma		Non-invasive lobular neoplasia:<br>– lobular carcinoma in situ NOS<br>– lobular carcinoma in situ, pleomorphic<br>Papillary neoplasms:<br>– ductal carcinoma in situ, papillary<br>Encapsulated papillary carcinoma:<br>– solid papillary carcinoma in situ<br>Ductal carcinoma in situ:<br>– ductal carcinoma in situ, non-infiltrating, NOS	Invasive breast carcinoma:<br>– infiltrating duct carcinoma NOS<br>– oncocytic carcinoma<br>– lipid rich carcinoma<br>– glycogen rich carcinoma<br>– sebaceous carcinoma<br>– lobular carcinoma NOS<br>– tubular carcinoma<br>– cribriform carcinoma NOS<br>– mucinous adenocarcinoma<br>– mucinous cystadenocarcinoma NOS<br>– invasive micropapillary carcinoma of breast<br>– metaplastic carcinoma NOS<br>Rare and salivary gland type tumors:<br>– secretory carcinoma<br>– acinar cell carcinoma<br>– mucoepidermoid carcinoma<br>– polymorphous adenocarcinoma<br>– adenoid cystic carcinoma<br>– tall cell carcinoma with reversed polarity<br>Neuroendocrine neoplasms:<br>– neuroendocrine tumor, NOS<br>– neuroendocrine tumor, grade 1<br>– neuroendocrine tumor, grade 2<br>– neuroendocrine carcinoma NOS,<br>– neuroendocrine carcinoma, small cell

				–neuroendocrine carcinoma, large cell<br>Epithelial - myoepithelial tumors:<br>–adenomyoepithelioma with carcinoma<br>–epithelial-myoepithelial carcinoma<br>Papillary neoplasms:<br>–encapsulated papillary carcinoma with invasion<br>–solid papillary carcinoma with invasion<br>–intraductal papillary adenocarcinoma with invasion
**Mesenchymal tumors**				
Vascular tumors:<br>–angiomatosis<br>Other mesenchymal tumors and tumor-like conditions:<br>–pseudoangiomatous stromal hyperplasia	Vascular tumors:<br>– hemangioma NOS<br>– postradiation angiosarcoma<br>– atypical vascular lesion<br>Fibroblastic and myofibroblastic tumors:<br>– nodular fasciitis<br>– myofibroblastoma<br>Peripheral nerve sheath tumors:<br>– schwannoma NOS<br>– neurofibroma NOS<br>– granular cell tumor NOS<br>– adipocytic tumors;<br>– lipoma NOS<br>– angiolipoma NOS<br>Smooth muscle tumors:<br>– leiomyoma NOS	Fibroblastic and myofibroblastic tumors:<br>–desmoid type fibromatosis<br>–inflammatory myofibroblastic tumor		Vascular tumors:<br>–postradiation angiosarcoma<br>–angiosarcoma<br>Peripheral nerve sheath tumors:<br>–granular cell tumor, malignant
**Fibroepithelial tumors**				
–hamartoma	– fibroadenoma NOS	–phyllodes tumor,		–phyllodes tumor, malignant

	– phyllodes tumor, benign	borderline<br>– phyllodes tumor NOS		– smooth muscle tumors<br>– leiomyosarcoma NOS<br>– adipocytic tumors<br>– liposarcoma NOS
**Tumors of the nipple**				
	– nipple adenoma<br>– syringoma NOS			– Paget disease of the nipple
**Malignant lymphoma**				
				– diffuse large b cell lymphoma NOS<br>– Burkitt lymphoma NOS/acute leukemia, Burkitt type<br>– breast implant associated anaplastic large cell lymphoma<br>– mucosa associated lymphoid tissue lymphoma<br>– follicular lymphoma NOS
**Tumors of the male breast**				
– gynecomastia			– in situ carcinoma	– invasive carcinoma

NOS - not otherwise specified.
